# Concepts in interaction: social engagement and inner experiences

**DOI:** 10.1098/rstb.2021.0351

**Published:** 2023-02-13

**Authors:** Anna M. Borghi, Albertyna Osińska, Andreas Roepstorff, Joanna Raczaszek-Leonardi

**Affiliations:** ^1^ Dynamic and Clinical Psychology, and Health Studies, Sapienza University of Rome, 00185 Rome, Lazio, Italy; ^2^ Institute of Cognitive Sciences and Technologies, National Research Council, 00185 Rome, Lazio, Italy; ^3^ Faculty of Psychology, University of Warsaw, Warsaw, Poland; ^4^ Interacting Minds Center, Aarhus University, Aarhus, Denmark

**Keywords:** social interaction, interaction with oneself, concepts, categorization, embodied cognition, grounded cognition

## Abstract

This theme issue aims to view the literature on concepts through a novel lens, that of social interaction and its influence on inner experiences. It discusses unsolved problems in literature on concepts, emphasizing the distinction between concrete versus abstract concepts and external versus internal grounding. This introductory article reflects the two research streams that the theme aims to bridge—in this area, the dimension of embodied interaction with others and how this influences the interaction with ourselves is still underexplored. In the first part, we discuss recent trends in social cognition, showing how interacting with others influences our concepts. In the second part, we address how social interactions become part of our inner world in a Vygotskian fashion. First, we illustrate how interoception, emotion and metacognition are connected with concepts and knowledge. Second, we deal with how language, in both its outer and inner form, can empower cognition and concepts. We also briefly describe how novel experimental and computational methods contribute to investigating the online use of concepts. Overall, this introductory article outlines the potentialities of an integrated and interactive approach that can give new, fresh life to a topic, that of concepts, which lies at the root of human cognition.

This article is part of the theme issue ‘Concepts in interaction: social engagement and inner experiences’.

## 1. Introduction

Concepts represent the building blocks of knowledge; they are crucial for thinking, inferring and interacting with the environment. They are the ‘glue’ that connects our past, present and future experience [1]. Concepts are typically investigated in two ways: in their relation to categorization—they can be considered as the cognitive and mental aspects of categories—and as instruments of thought [2,3]. In this theme issue, we address concepts with a multifaceted approach: in their link with categorization, as means for thinking, and as ‘architectures in the dynamic flow of situated language use’ [4]. The introductory part of the theme issue is dedicated to discussing what concepts are, their function, and their relationship with language, and outlining an integrated approach that bridges the dimensions of embodied and linguistic interaction with others [5].

Literature on concepts has a long tradition, but many issues are still open. When forming concepts, we abstract from single instances and experiences. Language concurs with this process, helping to shape categories. Scholars struggle to understand the mechanisms underlying abstraction and its relationship with abstractness, i.e. the characteristic of abstract concepts such as ‘think’ and ‘phantasy’. In this theme issue, we focus both on the social bases for abstraction processes (e.g. [6]) and on how we develop the capability for abstractness, i.e. to form and use abstract concepts [7] (e.g. [8,9]). Embodied and grounded approaches to cognition have successfully underlined the role of sensorimotor processes and, recently, of interoception for concepts [10–12]. Several approaches have underlined the importance of language for conceptualization and especially for abstractness. Distributional semantics views have shown how linguistic associations help capture meaning (e.g. [13–15]), and influential models have pointed out that syntax is crucial to the development of concepts, especially abstract ones [16]. Recent developments have gone a step further. They have highlighted the importance of linguistic experience and social interaction for conceptual acquisition, representation, and use [17–26], intending language as a special mode of being in the world. Our theme issue reflects these fresh developments, outlining the emerging trends of studying concepts in *situated interactions*. Specifically, the first section focuses on concepts in situated interaction and the second section on how social interaction influences inner cognition.

The first emerging trend concerns the crucial role *social interaction* plays in cognition. Aside from the obvious social origin of language [27], and the social constitution of the environment since early cognitive development [5], the awareness that many phenomena are interactively built grounds the increasingly widespread second-person neuroscience [28,29]. In research on language use, cutting-edge approaches investigate dialogue as a form of joint action [30,31], and new tools allow researchers to explore the interactional dynamics underlying language use [32–35]. Studies show that interaction facilitates abstract thought and problem-solving [6,36,37] and reveal that emotions [38–42] and social interaction [43–45] are paramount for abstract concept acquisition and use.

The first section of the theme issue reflects these new approaches. It includes studies and reflection on the very nature of concepts considered from the social-interactive perspective, with social interaction being a vital source for concepts and—in turn—concepts and language guiding social interaction. It addresses the relationship between concepts and social interaction from multiple viewpoints. Some papers adopt a developmental perspective. Others investigate the dynamics underlying conceptual use in interactive situations, i.e. during dialogue, while outsourcing our knowledge, and during collective problem-solving. The third group of papers outlines the neural underpinning of social concepts and the clinical implications of an approach that goes beyond single individuals.

A second emerging trend reflected in our theme issue concerns how different ways of *interacting with ourselves* potentiate our concepts and cognition. Language is a powerful instrument that enriches our cognitive abilities. Work on inner speech has recently had a novel impulse, showing that we use different kinds of inner speech—monologic and dialogic, condensed and expanded—and that inner speech influences and enhances our thinking processes [46,47]. In parallel, novel approaches have confirmed that language affects categories and fosters cognition [48–52], facilitating abstraction and abstractness. Aside from language, the interest in the body's role in cognition has received a new impulse from work on interoception, i.e. the sensitivity to inner bodily signals [53]. Evidence shows that concepts, primarily abstract ones, evoke interoceptive experiences [11,54].

The second part of our theme issue addresses how interacting with ourselves, either using language or considering bodily signals, potentiates cognition and impacts concepts. Importantly, the different roles sensorimotor and inner dimensions play, and their different weight, might help differentiate concepts into more abstract and more concrete ones [55]. Some papers focus on the role sensorimotor, interoceptive, and emotional aspects play in grounding concepts and on the importance of metacognition for the emergence and spread of abstract concepts; they adopt different methodologies, from computational models to the analysis of databases. Other papers focus more explicitly on language and its relationship with concepts, showing the importance of inner language, verbal labels and word associations for concrete and abstract concepts, and highlighting the role of language in enhancing cognition. Across the various sections, the theme issue offers many insights into the differences between kinds of concepts, from the significant distinction between concrete and abstract ones [2,8,9,56,57], to specific concepts like the religious [58], the social [59,60], the olfactory [61] and the emotional ones [38,42].

While our theme issue focuses on the investigation of concepts and categorization through the lens of social interaction, it also has implications in other areas, for example, for research on metacognition [3,9,61,62], and theoretical perspectives such as ecological psychology and extended cognition [5,63,64].

Our theme issue also has a variety of methodological implications. It offers hints for new methodological instruments that might allow us to investigate one of the most basic and, at the same time, sophisticated human processes, i.e. categorization, with novel methods. Some examples are simulations of the emergence of categories in individuals and populations, and new computational models (e.g. [57,62]), new ecological methods (e.g. [64]), new sophisticated data analysis techniques, including cross-linguistic analyses (e.g. [8,38,42]), new constructs and ways to investigate abstractness (e.g. [2,65]), and new neuroscience methodologies, including dual-person neuroscience (e.g. [29,60]). We also include a pledge to integrate qualitative microanalyses with quantitative methods [5]. In the next section, we will overview the theme issue, briefly describing the various sections in which it is organized and the contribution of the single papers.

## 2. Overview of this theme issue

The theme issue includes theoretical and research papers. The first section, *‘What's in a concept’*, is a general introduction to what concepts are. Adopting anthropological, philosophical and psychological perspectives, it focuses on concepts, abstraction, abstractness, and the difference between concepts and linguistic concepts. Owing to its introductory character, it includes four mainly theoretical papers.

*Enfield* [4], focusing on linguistic concepts, argues that attention to what they ‘stand for’—as is common in many semantic theories—ignores the processes that make such connections possible at all: the causal, eliciting power of linguistic concepts to generate interpretants in social situations. Enfield turns to semiotics to specify the two aspects that are inseparable for understanding the emergence and use of linguistic concepts: the object-axis and the interpretant-axis, in concert, are responsible for ascribing conceptual content to language. Concepts are ‘architectures in the dynamic flow of situated language usage’.

According to *Shea* [3], concepts are components of conscious thought that can be variously combined. In his paper, the author highlights that, while research on concepts typically focuses on the categorization of stimuli, we use concepts also to think about what to do, starting from thoughts rather than from external stimuli. To access this information, we run a simulation. Simulating cannot be simply equated with retrieving information from memory or inferring it by reasoning. Concepts should be seen as ‘plug & play devices’ allowing us to run simulations. Simulations are extremely effective because we can ‘unplug’ representations from the world and ‘plug’ them into simulations, allowing us to play with concepts offline.

In his paper, *Langland-Hassan* [2] focuses on some important terminological and methodological issues. Starting from discussing how concepts are typically conceived in different disciplines, he focuses on concepts in their relationship with categories. He then discusses current definitions of abstractness, intended as related either to the diversity of the perceptual features of the conceptual referents or to the detachment from sensory modalities (imperceptibility). He contends that these definitions are insufficient to account for the kind of abstraction people use in nonverbal tasks. He then presents a new construct, trial concreteness, based on visual similarity and common setting scores and demonstrates its validity in light of experimental evidence.

Finally, *Rączaszek-Leonardi & Zubek* [5] take a radical empiricist stance to understanding concepts. Following William James, they argue that concepts are possibilities for selection based on discovering new relations in the potential, latent perceptual organization. They underscore the possibility of direct perception of such relations and thus recognize the continuity between perception and conceptualization. Furthermore, they advocate restoring trust in first-person experiences as the most important anchor point for the relations that concepts are built on. In this way, they attempt to bridge recent research on concepts in cognitive science with the ecological and enactivist approaches, showing their compatibilities and complementarities.

Section 2, *‘Concepts and social interaction’*, addresses how concepts emerge from social interaction and how interaction influences their acquisition and use. It also outlines new instruments and methods that allow the study of concepts in social interaction. We chose this section to precede the following one, which focuses on interaction with ourselves thanks to instruments, such as language, learned and developed through interacting with others. This choice is motivated by the adoption of a Vygotskian perspective—according to Vygotsky, language is first developed socially, then it influences thought and inner processes, assuming the form of inner speech.

This section is divided into three parts—the first includes three papers adopting a developmental perspective, the second focuses on the dynamics of social interaction, while the third addresses how social concepts are represented in the brain and how the social dimension influences and constrains the clinical and psychiatric intervention.

The subsection '*Concepts and social interaction: developmental aspects*' includes three papers on conceptual development—a review and two research papers.

*De Felice* *et al.* [66] tackle the issue of concept acquisition with others, highlighting the importance of social context for learning in children and adults. The paper is a review of the behavioural and neuroimaging research on social human learning, with an aim to aid the development of novel research methodologies. They urge for the study of learning to return to its natural ecology, which is the social niche.

In their empirical paper, *Karmazyn-Raz & Smith* [64] follow ecological psychology's call to ‘ask not what's in your head but what your head is in’ and take a close look at how the environment of early interactions is dynamically structured for a child. In naturalistic play situations, they demonstrate the usefulness of narration analysis methods to uncover the primary ‘data’ structures and how the children's and caregivers' experiences align. Novel methods, such as recurrence analysis and network analyses, reveal temporal statistics of human-generated events, which demonstrate patterns and coherence similar to narratives or stories. ‘Like words in a discourse, or characters in a story, toy selections cohere into an integrated experience.' From such patterning of learning experience, the authors draw conclusions about the nature of memory structures and processes.

In their paper, *Viertel et al.* [58] present a study focusing on a religious word, the word ‘mercy’, in which they show how children co-construct the word meaning together with their caregivers. Specifically, the authors investigate seven- to eight-year-old children while reading a book with their parents, examining the verbal behaviour of both children and parents. Next, the authors assess to what extent children comprehended the meaning of the abstract word with the help of picture cards. The authors analyse the caregivers' and the children's production during the reading and the comprehension situations. Specifically, they examine three dimensions they deem crucial for conceptual learning. These dimensions are the use of emotionally rich speech (revealed by prosody, emotionally valenced words, etc.), the adoption of the other's perspective (as appearing through voice modulations, mental state verbs, etc.), and the degree of active participation of the children in the interaction.

The subsection ‘*Dynamic aspects in concepts and social interaction: transmission, sharing, alignment*’ focuses on concepts and social interaction, highlighting the dynamic aspects that characterize their interaction. It includes three papers focusing on how people offload conceptual understanding onto other people, how social interaction stimulates abstraction in groups, and how we dynamically align with our interlocutors during dialogue.

*Andrade-Lotero et al.* [63] present an empirical study in which participants collaborate on a task, each of them possessing expertise that is complementary to the other's. Participants chose between using their own classification ability versus off-loading on their partner's ability, pooling their resources. However, the second strategy was chosen relatively rarely (40% of the time), pointing to the social costs that such a strategy incurs. This strategy indeed raised the rate of success in the task. Interestingly the self-assessment of understanding was higher in participants when they were assigned the role of an expert in a dyad. The findings deepen the understanding of the phenomenon of division of linguistic labour, i.e. constantly relying on the distributed nature of cognition and the contribution of others to understand and use concepts.

*Olsen & Tylen* [6] focus on social interaction's role in developing and using abstraction. They distinguish abstraction from abstractness, and intend abstraction as a form of generalization across various experiences that promotes flexible interactions with the environment. While abstraction is typically investigated as an individual process, the authors contend that social interaction might stimulate and enhance abstraction processes in groups of people. In addressing this topic, they show that social interaction might play a different role, depending on the task and individual differences, suggesting that it facilitates access to information in the case of convergent thinking and enhances exploratory search in the case of divergent thinking. They also highlight potential limitations of excessive alignment when group members are too similar and discuss the benefits of the diversity of the group members.

*Gandolfi et al.* [31] investigate dialogue, considered the most effortless way speakers develop a common way to conceptualize the world, providing rich examples of linguistic interactions. They contend that speakers understand each other when they reach alignment; linguistic alignment is typically the sign of conceptual alignment, i.e. the development of common conceptualizations. To achieve alignment, speakers work on dialogue as a shared workspace. In this context, both metacognitive and social cognition abilities are crucial in order to monitor and control the contribution of each interlocutor. The authors also focus on abstract concepts, arguing that reaching alignment on abstract concepts might require linguistic negotiation. By contrast, it might be less the case for concrete concepts, the referents of which might be visible to both interlocutors.

The subsection ‘*Social concepts and interacting brains*' focuses on social concepts and their brain representation and the implications of focusing on social interaction for clinical research and the conceptualization of psychiatric disorders.

*Pexman et al.* [59] suggest that socialness is a key information type that may be a means to ground and organize abstract concepts. However, there currently is no common definition of ‘socialness’, though it is required to compare theories of conceptual representation, as well as evaluate and refine them. The authors present evidence from a large-scale rating study showing ‘socialness’ to be a distinct dimension of lexical-semantic knowledge of word meaning, distinguishable from other dimensions such as concreteness or valence.

*Lopes da Cunha et al.* [60] use a novel paradigm in which they combine a novel naturalistic text-reading paradigm, a relevant atrophy model, and functional magnetic resonance imaging (fMRI) to investigate the comprehension of social and non-social texts in patients with cerebellar ataxia (CA) and controls. They found that CA patients were impaired in grasping social but not non-social concepts. This study demonstrates for the first time an important role of the cerebellum in the conceptual construal of events involving social interaction between two people.

In their paper, *Bolis et al.* [29] illustrate the notion of interpersonal attunement, which allows the formation of social expectations in order to successfully interact with others and with oneself. They identify the predictive processing approach as a way to better capture these interactive dynamics. Then they discuss the dialectical misattunement hypothesis, showing how psychopathology can be seen as a disorder related to social interaction, i.e. a mismatch of interpersonal expectations, which can lead to a disruption of communication and induce social isolation. In this framework, they highlight how mental health is inextricably connected with social interaction and contend that this link should be recognized in the clinical area, leading to the development of forms of inter-personalized psychiatry.

The third section, ‘*Concepts and interaction with ourselves*’, focuses on how social interaction influences inner experiences and how we entertain a dialogue and interact with ourselves. The first part deals with grounding concepts in multimodal sensorimotor experiences and inner experiences. Indeed, we can interact with ourselves in various ways: developing the sensitivity to understand our bodily signals (interoception) or monitoring our own thoughts (metacognition). The second part focuses on interaction with ourselves thanks to instruments, such as language, acquired through interacting with others. We can use these socially developed instruments either internally (e.g. inner speech) or not (e.g. overt speech) as a guide for our cognition.

The subsection ‘*Concepts and sensorimotor and inner experiences*' focuses on the grounding of concepts and highlights the relevance of sensorimotor and inner experiences (interoception, metacognition) for different kinds of concrete and abstract concepts.

The paper by *Banks & Connell* [8] focuses on the sensorimotor grounding of categories differing in abstractness. Using abstract and concrete concepts and ratings taken from the Lancaster sensorimotor norms, they compare sensorial and action experiences across the two domains. They show that both kinds of categories are grounded in sensorimotor experiences, even if to a different extent, and that different sorts of sensorimotor experiences weigh differently: abstract categories evoke more interoception, hearing, movements of the mouth and head and of the torso and foot/leg. By contrast, concrete categories evoke more frequently haptic experiences, hand/arm movements, vision, smell and taste. The authors also show that abstract categories' sensorimotor grounding is more diffuse compared with concrete concepts and that the role of different modalities varies depending on the kind of concrete and abstract concepts. They conclude by highlighting the limitations of a dichotomic vision of abstract and concrete concepts.

*Barca et al.* [38] introduce a novel methodology to study emotional concepts. The authors note that emotional concepts are not processed in the void but in the context of other emotional stimuli and factors characteristic of an individual (e.g. physiological or interoceptive state, emotional disposition), all of which impact emotion perception and representation. They describe a new similarity index for emotional concepts based on decision uncertainty in an ambiguous context (measured through mouse-tracking). The authors then construct a topographical map of emotional concepts, sensitive to individual variations in affectivity and physiological measures.

In his paper, *Winter* [42] addresses claims of the influential affective embodiment account, according to which abstract concepts are grounded in emotions. He shows that abstract concepts typically obtain more strongly negative or positive ratings than concrete ones across languages as diverse as Mandarin Chinese, Polish, Dutch and Spanish. However, the effect is driven by a small set of abstract concepts, i.e. the emotional ones. This result questions the crucial role of emotions in characterizing all abstract concepts, suggesting both that it is essential to analyse differences across their subkinds and that multiple dimensions concur in their representation.

In her paper, *Deroy* [61] deals with a specific concept, i.e. olfaction. Olfaction concepts are intriguing because they are at the border between abstract and concrete concepts and vary in the amount of sensory experience. She outlines the puzzle research on olfaction deals with: abstract terms referring to olfaction are only a few, and most languages focus instead on the source of the smell (e.g. people use ‘the smell of lemon’ instead of ‘acrid’ or ‘fruity’). She contends that the puzzle should be reversed; given the characteristics of olfaction and the fact that commonalities between smells are not obvious, it is surprising that abstract terms are so many in this domain. She discusses two possible reasons for the extensive use of abstract concepts in this domain, addressing their potential communicative and social benefits. The first is that abstract concepts make people feel less authoritative and defer more to others; hence there would be a benefit in terms of social cohesion. The second is that converging on abstract terms would be easier. Hence, among the adaptive advantages that the use of abstract terms can provide, she identifies the social feedback received by others, which could increase people's confidence that their communicative intention has been understood. Both hypotheses strongly highlight the link between concepts, metacognition, and social interaction.

*Mannella & Tummolini* [62] set off to individuate processes that can be responsible for internal motivation for forming the concepts in the first place. They present a computational model, which is anchored in the physical environment but crucially uses an internal signal of the agent's sensorimotor coherence from different modalities: vision, touch, proprioception and action. The process of conceptual emergence assumes mapping the various sensorimotor experiences on a low-dimensional common space, which allows their alignment. This internal coherence becomes a source of conceptual modification as important as the history of interaction with external events. In this grounded processual approach, concept learning is understood as competence acquisition through this increasing convergence, where a system's memory is a reactivation of the states in the common space. State-of-the-art computational modelling is used to prove the coherence and feasibility of the theory

The subsection ‘*Concepts and the power of language*’ focuses on how inner and overt language enhance categorization. The three papers—one theory/ideas paper, one research paper, and one opinion paper—address the role of language for concrete and abstract concepts.

The paper by *Borghi & Fernyhough* [9] focuses on the role inner speech plays during conceptual acquisition and use. They contend that inner speech might be particularly crucial for abstract concepts owing to their complexity and the following uncertainty they generate. Various kinds of inner speech might be crucial for different processes, such as conceptual acquisition and use and different kinds of abstract concepts. They propose the notion of inner social metacognition—when processing abstract concepts, people internally monitor their knowledge and search for the possible meaning, for example, using dialogic inner speech. They also outline research lines that might emerge on the relationship between inner speech and abstract concepts.

In their paper, *Liu & Lupyan* [65] investigate how people evaluate similarities across different semantic domains, such as animals and jobs. One of the main reasons why studying cross-domain alignment is interesting is that it can be informative as to how people represent concrete and abstract concepts. For example, do people represent concrete concepts in terms of their sensorimotor aspects? The authors present three experiments, two free-response tasks and a goodness rating task, in which they demonstrate that people easily create mappings and converge in their mappings. Significantly, they form mappings and evaluate cross-domain similarities relying on abstract dimensions such as valence, activity, potency and gender.

*Dove* [56] puts forward a novel perspective on embodied cognition in which the language system is conceived as a form of embodiment. He draws evidence from the research on iconicity, the influence of linguistic labels on reasoning, the structure of the symbol system, inner speech and neuroimaging. Specifically, he discusses how iconicity contributes to concept acquisition and use, how linguistic labels influence concepts, and how relations among words can capture some conceptual content, and describes the role inner speech can play in accessing the content of our thoughts. Overall, his contribution strongly emphasizes the role of language in conceptualization.

## 3. Conclusion

This is the first theme issue that focuses on concepts highlighting their dependence on social interaction, and on interaction with oneself. The novel lens through which we view concepts emphasizes their role not only in action but also in co-action with others. Although recent years have brought a surge of research on how concepts are embodied, the dimension of embodied interaction with others and how this interaction might influence our interaction with ourselves is still underexplored. Thus, a synthesis of classical and embodied views along this dimension is urgently needed. This perspective bridges two broad and successful research lines: the first on social cognition and the second on categorization and abstraction. We hope that the cross-disciplinary character of the theme will be able to go beyond the two broad communities investigating concepts and social cognition, underscoring the social nature of categorization and abstraction, influencing research on interoception, inner speech, language acquisition, cross-cultural studies and human–artificial intelligence (AI) interaction. Neural network simulations can provide new insights into understanding how the processes of abstraction and abstractness unfold [67]. These insights might be crucial for the future development of artificial intelligence systems, including robots able to flexibly adapt to different physical and social environments. With the combinations of studies on conceptual development and use in adults, we seek to offer a new, integrated perspective, which we hope will contribute to rendering an approach that is solidly based on the study of development, appealing and enriching the study of education and teaching of social implications. We also hope that the integrated study of concepts and interaction, with others and with oneself, will provide new insights and instruments for research in the clinical area, with patients and atypically developing children. Overall, we believe that an interactive approach like the one we present can give new, fresh life to a topic, that of concepts, that has interested scholars for ages.

## Data Availability

This article has no additional data.
