# Correction to: ‘Concepts in interaction: social engagement and inner experiences’ (2022) by Borghi *et al.*

**DOI:** 10.1098/rstb.2023.0001

**Published:** 2023-04-10

**Authors:** Anna M. Borghi, Albertyna Osińska, Andreas Roepstorff, Joanna Raczaszek-Leonardi

*Phil. Trans. R. Soc. B* **378**, 20210351. (Published online 26 December 2022.) (https://doi.org/10.1098/rstb.2021.0351)

In the original version of this article, the title contained an error with the inclusion of the word ‘Editorial’.

This has now been corrected on the publisher's website.

